# Early Change in Platelet Count and MPV Levels of Patients Who Received Hemodialysis for the First Time: Mogadishu Somalia Experience

**DOI:** 10.1155/2022/1503227

**Published:** 2022-06-30

**Authors:** Oznur Sari, Ahmed Muhammad Bashir

**Affiliations:** ^1^Department of Internal Medicine, Department of Health Services, General Directorate of Public Hospitals, Ministry of Health, Ankara, Turkey; ^2^Department of Internal Medicine, Mogadishu Somalia Turkey, Recep Tayyip Erdogan, Training and Research Hospital, Mogadishu, Somalia

## Abstract

**Introduction:**

Mean platelet volume (MPV) is a marker used to assess the platelet' size and is also an indicator of platelet reactivity and prothrombotic status.

**Objective:**

In this study, we aimed to determine the relationship between MPV and biochemical parameters in patients who had received hemodialysis (HD) for the first time and then in respect of those same patients after their fourth HD.

**Method:**

151 HD patients were enrolled in this study. Patients were eligible for inclusion if they had received their first HD session during this study protocol. Prehemodialysis blood samples were taken. Most laboratory values, including mean platelet volume (MPV) level and platelets (PLT) count, were measured before the first HD and after the fourth HD session for each patient.

**Results:**

Among the patients in our study, the mean age profile of the male patients (*n* = 103; 68.2%) was found to be higher than that of the female patients (*n* = 48; 31.8%) (53.62 ± 18.19 vs. 46.17 ± 17.9 years) (*p* = 0.019).In the patients' laboratory results after the fourth HD session, MPV, MPV/Plt, and Na values had increased to those after the first HD session (*p* < 0.001). When age and gender status were taken into account, the level of weak positive correlation with white blood cell count (WBC), neutrophil, and red cell distribution width (RDW) was found, while the weak negative correlation with platelet to lymphocyte ratio (PLR) was found (*p* < 0.001).

**Conclusions:**

In our study, we found that increase in MPV and MPV/PLT levels was significant in the fourth HD session of patients with CKD. It is also debatable that there are findings indicating an increase in platelet reactivity in the first weeks of the onset of HD. This could be an early indicator of the early prevention of cardiovascular diseases.

## 1. Introduction

A preprint has previously been published at Research Square [1]. Chronic kidney disease (CKD) is a recognised public health problem, with mortality rates exceeding those in the general population. For dialysis patients, annual mortality stands at 21.2%. While many organ systems are affected in the uremic process, cardiovascular disease (CVD) mortality is responsible for 45% of all mortality in dialysis patients [2].

The literature indicated that the start of dialysis and subsequent initial months are associated with increased mortality [3–6]. For patients with chronic kidney disease (CKD), these first months on hemodialysis (HD) have been shown to be a high-risk period for mortality. However, there is a relative paucity of data regarding cardiovascular event (CVE) rates following HD initiation. In particular, it is unclear whether the time to event admits of any variation with regard to CVE risk factors give evidence of any difference for both early and later dialysis periods.

Mean platelets volume, which a hematological analyzers can easily evaluate, is a useful marker of both platelet functions and activation. As a parameter, MPV indicates platelet activation and has been the subject of research in various patient groups with atherosclerosis [7, 8]. Larger platelets with metabolic and enzymatic properties are more active, and in a recent meta-analysis, it has been argued that this increased activity serves as a predictive biomarker for CVD [9].

However, the relative paucity of studies on the uremic population is problematic. İn this study, we aimed to find out the platelet activities during the initial stages of hemodialysis and the relationship between MPV and MPV/PLT with biochemical parameters in patients who had received HD for the first time and then in respect of those same patients after their fourth HD. The increased platelets activity can be used as an early indicator for cardiovascular prevention.

## 2. Materials and Methods

### 2.1. Study Design and Setting

This cohort cross-sectional study was carried out at Mogadishu Somali Turkey Training and Research Hospital in Mogadishu, the capital of Somalia, which is the largest training and research hospital in the country.

### 2.2. Inclusion Criteria

All adult patients who received their first hemodialysis session during this study protocol between October 2018 and July 2019. Total of 151 patients who fulfilled the eligibility criteria have been included in the study.

### 2.3. Exclusion Criteria

Patients with malignancy, on routine hemodialysis, with a history of recent trauma, surgery, or burns, with the evidence of acute infection, with a history of chronic liver disease and history of aspirin use, were excluded from the study.

### 2.4. Data Collection

The ethical approval was obtained from Institutional Review Board (IRB) of Mogadishu Somali Turkey, Recep Tayyip Erdogan, Training and Research hospital, and informed consent was taken from the patients.

Demographics such as age and gender were obtained from medical records. Routine biochemical tests were performed with an autoanalyzer (Mindray BS-400 Clinical Chemistry Analyzer). Parameters such as white blood cell (WBC) count, MPV, platelet count, and hemoglobin (Hb) were obtained from blood samples with an autoanalyzer (Sysmex XN-1000 Sysmex Corporation, Kobe, Japan).

Prior to hemodialysis, blood samples were obtained for measurement. Again, prior to the first HD session, MPV-level and platelet count among other laboratory values were measured for each patient, with these repeated following the fourth HD session. The interval between the time blood samples was drawn, and their subsequent analysis was less than 30 minutes.

### 2.5. Hemodialysis

All patients underwent native hemodialysis with a temporary jugular catheter using the Fresenius 4008S dialysis machine. The first HD session was 2 hours, and the following HD sessions were 4 hours duration each. Each hemodialysis session utilized a dialyzer with a blood flow rate of 250 to 300 ml/min, where the dialysate flow was 500 ml/min.

### 2.6. Data Analysis

We used SPSS 25.0 (IBM Corporation, Armonk, New York, United States) and PAST 3 (Hammer, Ø., Harper, D.A.T., Ryan, P.D. 2001. Paleontological Statistics) programs with regard to variable analysis. The Shapiro–Wilk Francia test evaluated the conformity of the univariate data to normal distribution. In contrast, the Mardia (Doornik and Hansen omnibus) test evaluated the conformity of multivariate variables to normal distribution. Comparison of the two independent groups according to the quantitative data was conducted via the independent sample *t*-test in conjunction with the Bootstrap results. The Bootstrap results were also employed with the paired-sample *t*-test.

The Wilcoxon signed-rank test, however, was used with the Monte Carlo simulation with respect to the comparison of the two quantitative dependent variables' repetitive measurements. The analysis of the correlation between the variables after controlling for the effects of age and gender was undertaken via the partial correlation test, with the analysis of the correlation between the variables before controlling for the effects of age and gender conducted via Kendall's Tau-b test. The quantitative data were set out in the tables as mean ± SD (standard deviation) (minimum/maximum) and median (1st quartile/3rd quartile), while the categorical variables were *n* (%). Analysis of the variables was conducted at the 95% confidence level, and a *p* value of less than 0.05 was held to be significant.

## 3. Results

### 3.1. Demographic Variables and Laboratory Results

Among the patients in our study, the mean age profile of male patients (*n* = 103; 68.2%) was found to be higher than that for female patients (*n* = 48; 31.8%) (53.62 ± 18.19 versus 46.17 ± 17.9 years) (*p* = 0.019) (Table 1).

Laboratory results of patients after the fourth HD session showed that MPV, MPV/PLT, MCV, Hb, HCT, and Na values had increased compared to before the first HD session (*p* < 0.001) (Table 2). As expected, a significant decrease was noted in BUN, creatinine, and *K* values (*p* < 0.001). There was no significant change in Ca, LYM, RDW, and PDW values (*p* > 0.05) (Table 2).

### 3.2. Relationship with MPV Change

Having controlled for the effects of age and gender, we found the level of positive correlation with WBC, NEU, and RDW to be low, while we found the level of negative correlation with PLR to be low (*p* < 0.001). Where age and gender effects were not controlled for, the level of weak positive correlation detected between MPV and RDW was found. A weak negative relationship was found between MPV and PLT, PDW, and PLR (*p* < 0.001) (Table 3).

## 4. Discussion

Mean platelets volume and various inflammatory clinical states have been the subject of a number of investigations. Significant changes in platelet reactivity and platelet levels are observed in patients with a diagnosis of CKD [10]. In chronic inflammatory conditions such as chronic kidney disease, platelet parameters were seen to differ significantly and were thus more marked in the state of HD. In our study, a slight increase in MPV levels was noted in patients in the first week of HD contrary to what is expected in the literature [11, 12].

The higher prevalence of CKD and HD in males is commonly observed, but its explanation is not fully understood. It could be due to the larger propensity for CKD to progress to ESRD in males than in females. In the United States, the prevalence of ESRD was highest among people aged 65 to 74 years old until 2010, when the margin between those aged 75 and older began to close. While those ≥75 showed the highest incidence rate, ESRD prevalence was somewhat lower as a result of increased mortality in this cohort [13]. The region in which the patients included in our study live is among those countries where gross domestic product (GDP) levels are low according to World Bank criteria [14]. This may be attributed to the fact that the rate of male patients in our study is high, while ESRD patients' average age is lower than normal or high-GDP societies. Cultural differences in the Mogadishu community also contribute to difficulties experienced by women in gaining access to and benefitting from healthcare services.

However, the number of studies conducted regarding changes in MPV in patients with CKD is low. The relevant literature indicates the difficulties in elucidating the influence of multiple determinants on PLT and MPV in physiology and during disease state, principally as a result of the differing factors affecting platelet reactivity [10]. Nevertheless, the evaluation of this aspect of MPV within clinical practice should be the subject of additional research. The study conducted by Balcioglu and Kirlioglu [15] found that there is a negative correlation between MPV and number of hospital stays and total duration of hospital stays.

Activated large platelets have an important role in the pathogenesis of atherothrombosis and cardiovascular events (CVE). Platelets are not only constituent elements of thrombosis formation, but contribute to the induction of inflammation [16]. Recent research has also shown that with respect to larger platelets, platelet reactivity levels are greater [17]. In The Anglo-Scandinavian Cardiac Outcomes Trial (ASCOT), patients with end-organ damage and hypertension (HT) showed higher levels of MPV [16]. However, in a study by Bilen et al. [18], it was seen that with respect to increased inflammation in CKD patients, MPV cannot be shown to have a predictive value. It has also been argued that the larger MPV seen in CKD can be attributed to platelet swelling rather than platelet reactivity. More recently, in HD patients, it has been observed that there is a negative correlation between plasma HCO3– and MPV [19]. Turgutalp et al. [20] found in their preliminary study results that patient MPV values were higher in those diagnosed with diabetic nephropathy than those in the normal population.

Given the existence of a nonlinear inverse relationship between PLT and MPV, it has been suggested that the assessment of MPV should always be correlated with platelet count [21]. Other factors, which may be germane including age, gender, race, ethnicity, and lifestyle (including diet), while evidence would suggest that both MPV and PLT are also strongly influenced by genetic factors [22, 23]. However, research to date is sparse with respect to changes in platelet-size parameters in patients with renal failure and according to GFR. With respect to CKD patients, Ju et al. previously noted higher MPV values and a progressive decrease in both platelet count and PDW [24]. Heritability was 84% and of 75%, respectively, for PLT and MPV, a higher rate, attributed to genetic variations [25].

We think that this study is unique because it includes the results of ESRD patients living in Somalia, where there are significant difficulties in accessing healthcare facilities. In our study, changes in platelet volume and number in patients in the first week of HD were similar to those in the literature. We would argue that further studies are required to evaluate the effect of race, ethnicity, and demographic features on the results.

## 5. Conclusions

In our study, we found that increase in MPV and MPV/PLT levels was significant in the fourth HD session of patients with CKD. It is also debatable that there are findings indicating an increase in platelet reactivity in the first weeks of the onset of HD. This could be used as an early alarming feature for cardiovascular disease. In particular, CKD patients have a risk for cardiovascular event, so we recommend monitoring closely with other risk factors too.

### 5.1. Limitations

There are some limitations to our study. We could not obtain the demographic features, chronic diseases, and CKD etiologies from the file system because they had not been integrated into the registry system. For this reason, it was only possible to evaluate HD patients with measured laboratory tests. Another main limitation is that we did not include the cardiometabolic parameters in our study. Socioeconomic difficulties and cultural constraints preclude a ready solution to this problem.

## Figures and Tables

**Table 1:** Age and gender distributions of hemodialysis patients.

	Female (*n* = 48), mean **±** SD. (Min./Max.)	Male (*n* = 103), mean **±** SD. (Min./Max.)	Total (*n* = 151), mean **±** SD. (Min./Max.)	*P*
Age (year)	46,17 ± 17,90 (22/86)	53,62 ± 18,19 (18/88)	51,25 ± 18,37 (18/88)	**0.019**

Independent samples *t*-test (Bootstrap); SD., standard deviation; Min., minimum; Max., maximum.

**Table 2:** Change in laboratory tests before the first hemodialysis and after the fourth hemodialysis.

	Before 1^st^ HD (*n* = 151) mean **±** SD.	After 4th HD (*n* = 151) mean **±** SD.	*P*
MPV (fL)	9,86 ± 1,49	10,06 ± 1,47	**0.045**
MCV (fL)	82,04 ± 5,88	83 ± 4,86	**0.008**

	Median (Q1/Q3)	Median (Q1/Q3)	
Urea (md/dL)	231 (180/309)	87 (62/112)	**<0,001**
Creatinine (mg/dL)	11,70 (7,53/18,26)	4,99 (3,43/6,96)	**<0,001**
Sodium (meq/L)	134 (128/139)	136 (133/140)	**<0,001**
Potassium (meq/L)	4,70 (3,99/5,59)	3,83 (3,32/4,33)	**<0,001**
Total protein (g/dL)	6,40 (5,50/7)	5,80 (5,30/6,50)	**<0,001**
Albumin (g/dL)	3,60 (2,90/4,10)	3,20 (2,70/3,50)	**<0,001**
Calcium (mg/dL)	8,10 (7,60/9)	8,20 (7,50/8,90)	0.896
WBC (^*∗*^10^3^)	10,77 (7,56/16,28)	8,25 (6,10/11,85)	**<0,001**
Neutrophil (^*∗*^10^3^)	8,10 (5,25/13,85)	5,80 (3,90/9,31)	**<0,001**
Lymphocyte (^*∗*^10^3^)	1,03 (0,76/1,58)	1,04 (0,71/1,52)	0.962
Hb (g/dL)	8,50 (7,30/9,90)	9,20 (8,30/10,10)	**0.004**
HCT (%)	24,90 (20,70/28,60)	26,70 (24,50/28,60)	**0.002**
RDW-CV (%)	14,60 (13,40/16,30)	14,60 (13,50/16,10)	0.052
Platelets (^*∗*^10^3^)	231 (171/315)	193 (140/260)	**<0,001**
PDW (%)	12,60 (10,40/16,20)	12,40 (10,20/16)	0.760
NLR	7,93 (4,76/14,48)	5,64 (3,52/11,33)	**<0,001**
PLR	215,12 (134,21/355,77)	185,71 (128,10/272,86)	**0.002**
MPV/PLT	0,04 (0,03/0,06)	0,43 (0,33/0,58)	**<0,001**

Paired-sample *t*-test (Bootstrap), Wilcoxon sign test (Monte Carlo); Q1, first quartile; Q3, third quartile; SD., standard deviation; WBC, total leucocyte; Hb, hemoglobin; HCT, hematocrit; RDW-CV, red blood cell distribution width-coefficient variation; NLR, neutrophil to lymphocyte ratio; PLR, platelet to lymphocyte value; MPV, mean platelet volume; PLT, platelets; fL, femtoliters; mg, milligram; L, liter; g, gram.

**Table 3:** Correlation of MPV exchange with other laboratory tests.

Difference MPV	Controlled for age and gender		Not controlled for age and gender	
	*r*	*P* ^pc^	*r*	*P* ^kc^
MCV	−0.020	0.811	−0.005	0.929
Urea (md/dL)	−0.054	0.510	−0.049	0.379
Creatinine (mg/dL)	0.032	0.701	−0.004	0.936
Sodium (meq/L)	0.007	0.936	0.059	0.296
Potassium (meq/L)	0.058	0.481	0.041	0.462
Total protein (g/dL)	0.048	0.558	0.007	0.894
Albumin (g/dL)	−0.056	0.499	−0.087	0.118
Calcium (mg/dL)	−0.013	0.876	−0.002	0.968
WBC (^*∗*^10^3^)	0.173	**0.035**	0.105	0.058
Neutrophil (^*∗*^10^3^)	0.171	**0.037**	0.101	0.067
Lymphocyte (^*∗*^10^3^)	0.152	0.065	0.085	0.125
Hb (g/dL)	−0.095	0.248	−0.053	0.335
HCT (%)	−0.118	0.153	−0.056	0.311
RDW-CV (%)	0.274	**0.001**	0.126	**0.024**
Platelets (^*∗*^10^3^)	−0.111	0.176	−0.139	**0.012**
PDW (%)	0.098	0.235	0.326	**<0,001**
NLR	−0.024	0.776	−0.005	0.933
PLR	−0.197	**0.016**	−0.159	**0.004**
MPV/PLT	0.149	0.069	0.093	0.092

^pc^ Partial correlation test; kc, Kendall's Tau-b test; r, correlation coefficient; WBC, total leucocyte; Hb, hemoglobin; HCT, hematocrit; RDW-CV, red blood cell distribution width-coefficient variation; NLR, neutrophil to lymphocyte ratio; PLR, platelet to lymphocyte value; MPV, mean platelet volume; PLT, platelets; fL, femtoliters; mg, milligram; L, liter; g, gram.

## Data Availability

The data used to support the findings of this study are available from the corresponding author upon request.
